# *Morinda officinalis* oligosaccharides attenuate mitochondria-associated ferroptosis via the NOX4/mitoGPX4 pathway in myocardial ischemia‒reperfusion injury

**DOI:** 10.3389/fcell.2025.1605513

**Published:** 2025-05-26

**Authors:** Yuqiong Chen, Yuan Tian, Bo Guan, Yiling Chang, Xiaopei Yan, Qi Song, Wenting Chen, Lin Chen, Wei Li, Wenjun Mao, Yan Zhang, Chao Chen, Su Li

**Affiliations:** ^1^ Department of Cardiology, The Affiliated Suzhou Hospital of Nanjing Medical University, Suzhou Municipal Hospital, Gusu School, Nanjing Medical University, Suzhou, China; ^2^ Department of Cardiology, Jinshan Hospital, Fudan University, Shanghai, China; ^3^ Department of Geriatrics, The Affiliated Suzhou Hospital of Nanjing Medical University, Suzhou Municipal Hospital, Gusu School, Nanjing Medical University, Suzhou, China; ^4^ The Affiliated Taizhou People’s Hospital of Nanjing Medical University, Taizhou, China; ^5^ Department of Respiratory Medicine, The Affiliated Suzhou Hospital of Nanjing Medical University, Suzhou Municipal Hospital, Gusu School, Nanjing Medical University, Suzhou, China; ^6^ Department of Anesthesiology, Xuzhou Central Hospital, The Affiliated XuZhou Hospital of Nanjing Medical University, Xuzhou, China; ^7^ Department of Cardiology, Zhongshan Hospital, Fudan University, Shanghai Institute of Cardiovascular Diseases, Shanghai, China; ^8^ National Clinical Research Center for Interventional Medicine, Shanghai, China

**Keywords:** *Morinda officinalis* oligosaccharides, myocardial ischemia-reperfusion injury, NOX4, GPx4, ferroptosis

## Abstract

**Aim:**

To explore the benefits of *Morinda officinalis* oligosaccharides (MOO) on ischemia-reperfusion (I/R) injury and the possible mechanisms involved.

**Methods:**

Myocardial I/R injury were induced by left anterior descending branch ligation. MOO pretreatment was given orally 2 weeks prior to ischemic treatment. Echocardiograms, biochemical parameters, and histological and immunohistochemical analyses were used to determine the benefits of MOO on myocardial I/R injury. Oxidative stress and ferroptosis were examined by biochemical parameters, Western blot, immunohistochemistry, and Tunel staining.

**Results:**

MOO improved cardiac function and reduced myocardial oxidative stress and ferroptosis, which was associated with the inhibition of NADPH Oxidase 4 (NOX4) expression. Whereas, the upregulation of NOX4 abolished the benefits of MOO. Furthermore, MOO enhanced mitochondrial superoxide dismutase 2 (SOD2) activity and stimulated the mitochondrial translocation of glutathione peroxidase 4 (mitoGPX4) by inhibiting NOX4. Mitochondria-specific GPX4 overexpression attenuated mitochondrial oxidative stress and suppressed mitochondria-associated ferroptosis in cardiomyocytes that suffered from hypoxia-reoxygenation (H/R) injury, even after NOX4 overexpression.

**Conclusion:**

These results indicate the beneficial effects of MOO on myocardial I/R injury by suppressing oxidative stress and mitochondria-associated ferroptosis through NOX4/mitoGPX4 pathway.

## 1 Introduction

Myocardial infarction (MI) is a prevalent disease conveying high mortality and morbidity (Mensah et al., 2023). Currently, reperfusion strategy is the most effective approach to salvage the ischaemic myocardium, yet it also causes additional injury known as ischemia–reperfusion (I/R) injury, which can account for up to 50% of the total infarct size (Yellon and Hausenloy, 2007). Several factors, such as calcium overload, oxidative stress, rapid restoration of intracellular pH, osmotic overload, changes in gap junction communication, and inflammatory signalling, are involved in the regulation of I/R injury (Algoet et al., 2023). Pharmacological interventions to reduce I/R injury are an important clinical gap that needs for future research. Therefore, it is essential to explore new pharmacological strategies to protect against I/R injury.

Redox homeostasis, a dynamic balance between the production and clearance of reactive oxygen species (ROS), plays a critical role in the development of I/R injury (Chen et al., 2021; Jiang et al., 2021). Oxidative stress and excessive ROS accumulation is caused by the disruption of this balance, which aggravates I/R injury to a great extent (Liu et al., 2024; Jin et al., 2017). ROS primarily originate from two primary pathways: the electron transport chain in mitochondria and mitochondrial enzymes, such as nicotinamide adenine dinucleotide phosphate (NADPH) oxidases, including NOX4 (Ren et al., 2023; Vendrov et al., 2023). The accumulation of excessive ROS results in damage to cellular structures and causes programmed cell death, such as apoptosis and ferroptosis (Hayes et al., 2020; Li et al., 2023; Park et al., 2021). NOX4 has been suggested to increase ROS production via the inhibition of GPX4 protein expression and therefore caused ferroptosis (Wu S. et al., 2024). Conversely, the antioxidant defence system, including SOD2-mediated enzymatic pathway, can scavenge excess ROS by the upregulation of GPX4 to restore cellular redox, thereby mitigating ferroptosis (Li et al., 2022). The effectiveness of therapies aimed to reduce ROS production via NOX4 inhibition or SOD2 activation has been demonstrated in alleviating cardiac I/R injury (Ma et al., 2021; Wu et al., 2023).

Ferroptosis is obviously distinct from apoptosis, necrosis, and autophagy, and is characterized by the accumulation of lipid ROS and free iron (Li et al., 2020; Yan et al., 2024). It has been implicated in various cardiac pathophysiological processes, including cardiac I/R injury and diabetic cardiomyopathy (Qu et al., 2024; Cai et al., 2023; Chen et al., 2023; Chen Y. et al., 2024). There are two main mechanisms involved in the suppression of ferroptosis, including the canonical glutathione peroxidase 4 (GPX4)-regulated ferroptosis inhibition pathway and GPX4-independent surveillance pathways, such as the ferroptosis suppressor protein 1 (FSP1) and DHODH (Bersuker et al., 2019; Wang et al., 2022). Prior evidence has demonstrated ferroptosis promoted myocardial I/R injury, and recognized GPX4-regulated pathway is of great important in this process (Yao et al., 2024). Recently, mitochondria-associated ferroptosis has been introduced to contribute to cardiovascular disease. Cytoplasmic ACSL4 translocated to mitochondria in diabetes, aggravated mitochondria-associated ferroptosis in endothelial cells, and thereby caused cardiovascular dysfunction in diabetic cardiomyopathy (Chen et al., 2023; Chen Z. et al., 2024). In addition, doxorubicin and trastuzumab suppressed mitochondria-localized GPX4, promote mitochondrial lipid peroxidation accumulation, and resulted in cardiomyocytes ferroptosis (Ye et al., 2023; Tadokoro et al., 2020). However, the pathways regulating mitochondrial GPX4 expression and the role of mitochondria-associated ferroptosis in myocardial I/R injury remain need further exploration.

*Morinda officinalis* oligosaccharides (MOO), a natural extract from the roots of M. officinalis, has been reported to have a variety of biological activities, including antidepressant, antioxidant, and anti-inflammatory activities (Zhang et al., 2018; Zhu et al., 2022). MOO has been suggested to defend against mitochondrial damage by reducing oxidative stress and promoting mitophagy (Yang et al., 2023). In addition, an experiment has shown that an inulin-type of oligosaccharides from *M. officinalis* protected against hypoxia-reoxygenation (H/R)-induced endothelial cell injury mainly through antioxidative effects and promotes angiogenesis by activating the PI3K/PKB/eNOS signalling pathway (Cha et al., 2019). However, it remains unclear whether MOO could improve myocardial I/R injury. Thus, this study was designed to investigate the beneficial effects of MOO on cardiac I/R injury and to explore the possible mechanisms in the context of oxidative stress and ferroptosis.

## 2 Results

### 2.1 MOO ameliorated myocardial I/R injury

**FIGURE 1:**
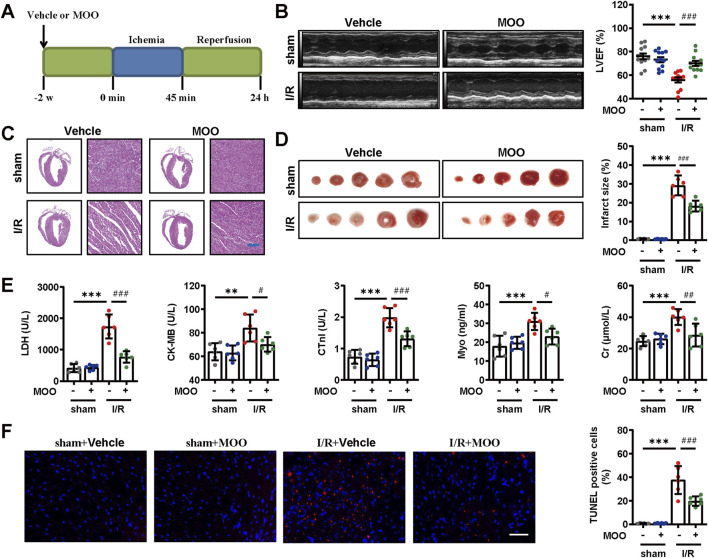
Effects of MOO pretreatment on cardiac function and morphology in I/R mice. **(A)** Protocol of MOO pretreatment and myocardial I/R procedure. **(B)** Representative images of echocardiographic analysis and statistical analysis of left ventricular ejection fraction (LVEF) in indicated groups (n = 12). **(C)** Representative images of HE staining of heart tissues. Scale bars = 40 μm. **(D)** Representative images of TTC staining and quantitative analysis of infarct size. **(E)** Effects of MOO on LDH, CK-MB, cTnI, Myo, and Cr levels in serum. **(F)** Representative images and quantitative analysis of TUNEL staining of heart tissues. Scale bars = 40 μm. Data are expressed as the mean ± standard error (SE) and analysed by one-way ANOVA followed by Tukey’s multiple comparisons test. *P < 0.05, **P < 0.01, ***P < 0.001 between sham and I/R groups. ^#^P < 0.05, ^##^P < 0.01, ^###^P < 0.001 between I/R and I/R + MOO groups. Four to twelve biological replicates were included in the experiment. (I/R, ischemia-reperfusion injury; MOO, *Morinda officinalis* oligosaccharides; LDH, lactate dehydrogenase; CK-MB, creatine kinase isoenzyme MB; cTnI, cardiac troponin-I antigen; ALT, alanine aminotransferase; Cr, creatine).

Whether MOO could attenuate myocardial tissue damage after I/R injury was not investigated previously. Therefore, mice were pretreated with MOO 2 weeks before I/R injury, and echocardiography and histopathological staining were used to evaluate the alterations in cardiac function and structure (Figure 1A). Compared with the sham group, I/R mice exhibited overtly compromised systolic function, as manifested by the declines in LVEF and LVFS, which was improved by MOO treatment (Figure 1B; Supplementary Figure S1A). In contrast to the regular and clear myocardial fibres and structures seen in the mice of the sham group, the myocardial fibres in the mice of the I/R group exhibited loose and irregular arrangements, with partial fragmentation (Figure 1C). Notably, MOO pretreatment mitigated these pathologic alterations (Figure 1C). Correspondingly, MOO pretreatment led to a reduction of infarct size in I/R mice (Figure 1D). In addition, we evaluated myocardial injury markers in the serum. I/R mice showed increased levels of LDH, CK-MB, CTnI, Myo, ALT, and Cr, when compared with mice from the sham group. However, these myocardial injury markers were obviously reduced by MOO treatment (Figure 1E). Furthermore, TUNEL analysis indicated that MOO pretreatment helped to reduce apoptotic cells in I/R mice (Figure 1F). These data support the cardioprotective effects of MOO on myocardial functional and structural damage caused by I/R injury.

### 2.2 MOO reduced mitochondrial oxidative stress after I/R injury

**FIGURE 2:**
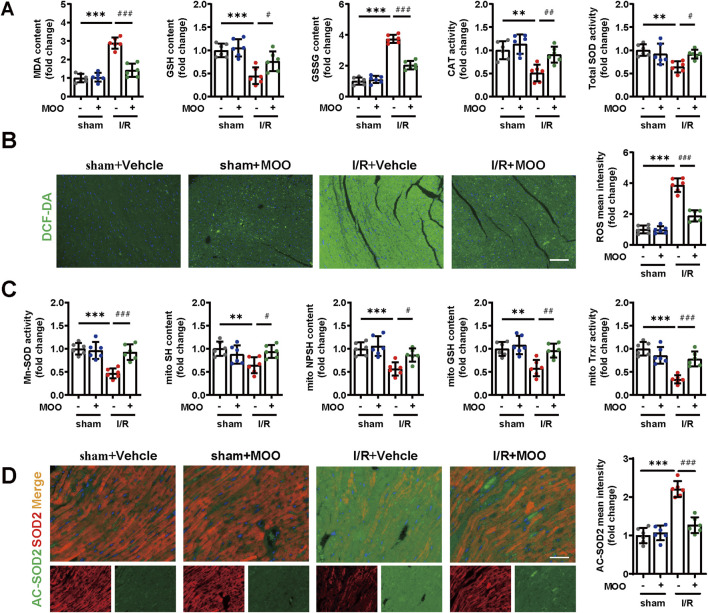
MOO pretreatment alleviated cardiac and mitochondrial oxidative stress in I/R mice. **(A)** The contents of MDA, GSH, and GSSG and the enzyme activities of CAT and SOD in heart tissues. **(B)** Representative images and quantitative analysis of cardiac ROS by immunofluorescence. Scale bars = 40 μm. **(C)** The contents of GSH, SH and NPSH and the enzyme activities of Mn-SOD and Trxr in mitochondria isolated from heart tissues. **(D)** Representative images and quantitative analysis of SOD2 and AC-SOD2 in heart tissues by immunofluorescence. Scale bars = 20 μm. Data are expressed as the mean ± standard error (SE) and analysed by one-way ANOVA followed by Tukey’s multiple comparisons test. *P < 0.05, **P < 0.01, ***P < 0.001 between sham and I/R groups. ^#^P < 0.05, ^##^P < 0.01, ^###^P < 0.001 between I/R and I/R + MOO groups. Four to six biological replicates were included in the experiment. (I/R, ischemia-reperfusion injury; MDA, malondialdehyde; GSH, glutathione; SOD, superoxide dismutase; ROS, reactive oxygen species; CAT, catalase; Trxr, thioredoxin reductase).

Given the contribution of oxidative stress in myocardial I/R injury, we investigated whether MOO pretreatment could mitigate I/R-induced oxidative stress. I/R stress led to elevated MDA and GSSG levels and decreased levels of CAT, GSH, and total SOD, the effects of which were reversed by MOO pretreatment (Figure 2A). Furthermore, MOO pretreatment inhibited the I/R-induced ROS accumulation (Figure 2B). Mn-SOD (SOD2) is a crucial mitochondrial antioxidant enzyme that plays an essential role in maintaining mitochondrial homeostasis (Chen et al., 2020). MOO was found to enhance Mn-SOD activity after myocardial I/R injury (Figure 2C). We therefore further evaluated markers of mitochondrial oxidative stress. I/R stress led to a decrease in CAT, GSH, TRXR, total SH, and NPSH levels in mitochondria, which was inhibited by MOO pretreatment (Figure 2C; Supplementary Figure S1B). The above results suggested that MOO pretreatment attenuated oxidative stress in mitochondria based on multiple markers.

Acetylation of SOD2 (AC-SOD2) results in the inactivation of SOD2, and we further quantified the protein levels of SOD2 and AC-SOD2. Immunofluorescence and Western blot analyses indicated that I/R stress led to decreased SOD2 levels and elevated AC-SOD2 levels, and these trends were reversed by MOO pretreatment (Figure 2D; Supplementary Figures S1C, D). Peroxisome proliferator-activated receptor-gamma coactivator-1alpha (PGC-1α) expression is a key index in the evaluation of mitochondrial function. The results showed that PGC-1α protein levels were decreased by I/R stress, whereas this decrease was inhibited by MOO pretreatment (Supplementary Figure S1D). These *in vivo* data demonstrate that MOO alleviated I/R-induced mitochondrial oxidative stress and dysfunction.

### 2.3 MOO reduced myocardial oxidative stress injury by targeting NOX4

**FIGURE 3:**
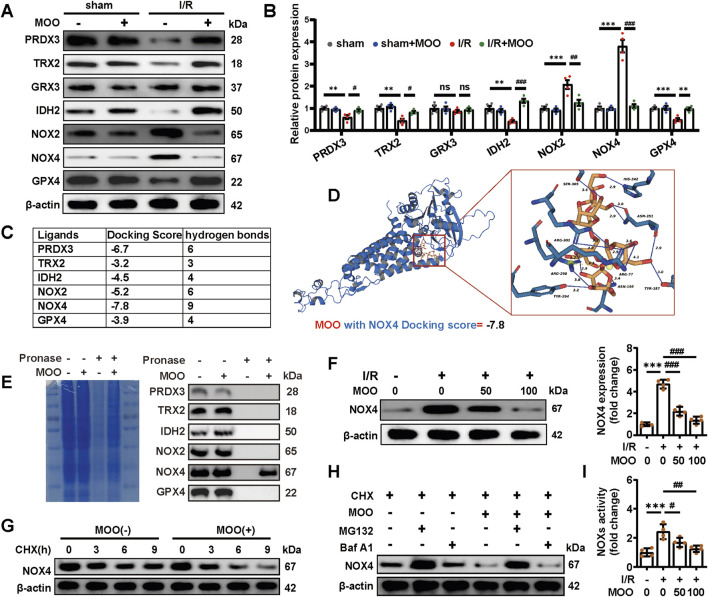
MOO pretreatment regulated oxidative stress by targeting NOX4. **(A, B)** The expression of oxidative stress-related proteins in heart tissue was detected by Western blot analysis and quantified. **(C)** The docking score and number of hydrogen bonds between MOO and ligands were performed and analysed using Schrödinger-Maestro and PLIP. **(D)** Molecular docking based on the chemical structure of MOO and the AlphaFold3 structure of NOX4 was shown. **(E)** DARTS assay was performed in NMVCs after MOO pretreatment for 24 h. The protein expression before and after pronase digestion was detected by Western blot. **(F)** MOO was added to NMVCs on an escalating dose and western-blots were used to examine NOX4 expression. **(G)** Cyclohexane (CHX) was added to NMVCs at indicated time, with or without MOO pretreatment. The expression of NOX4 was detected by Western blot. **(H)** CHX was added to NMVCs for 6 house, MG132 and Bafilomycin A1 (Baf A1) was used to inhibited protease and autophagy degradation of NOX4 respectively in indicated groups. **(I)** Statistic analysis NADPH oxidases (NOXs) activity. Data are expressed as the mean ± standard error (SE) and analysed by one-way ANOVA followed by Tukey’s multiple comparisons test. *P < 0.05, **P < 0.01, ***P < 0.001 between sham and I/R groups. ^#^P < 0.05, ^##^P < 0.01, ^###^P < 0.001 between I/R and I/R + MOO groups. ns, not significant. Four to six biological replicates were included in the experiment. (I/R, ischemia-reperfusion injury; MOO, *Morinda officinalis* oligosaccharides; NOX4, NADPH Oxidase 4; NMVCs, newborn murine ventricular cardiomyocytes).

We confirmed that pretreatment with MOO ameliorates I/R-induced oxidative stress in both myocardial tissue and mitochondria. To further evaluate the specific molecular mechanism by which MOO regulates I/R-induced myocardial oxidative stress, we examined the protein expression levels of a battery of mitochondrial oxidative stress-associated genes. The results suggested that I/R stress significantly reduced the protein expression levels of peroxiredoxin 3 (PRDX3), thioredoxin 2 (TRX2), isocitrate dehydrogenase 2 (IDH2), and GPX4 and increased the protein expression levels of NOX2 and NOX4, whereas these trends were reversed by MOO pretreatment (Figures 3A, B). To screen for potential downstream binder of MOO, we calculated the docking score and number of hydrogen bonds between MOO and ligands (Figure 3C). NOX4 was the protein with great potential presented by a docking score of −7.8 (Figures 3C, D). To further test the actual nature of the interactions, DARTS assay was performed in newborn murine ventricular cardiomyocytes (NMVCs) after MOO treatment for 24 h, and the results showed that NOX4 was the only protein that binds with MOO (Figure 3E). Then the myocardial I/R injury model was again established and pretreated with different concentrations of MOO. The results showed that MOO decreased NOX4 protein levels in a dose-dependent manner (Figure 3F). To further explore how MOO affected NOX4 expression, we added Cyclohexane (CHX) to NMVCs and found MOO accentuated the protein degradation of NOX4 (Figure 3G). Additionally, the protease inhibitor MG132, rather than autophagy inhibitor Bafilomycin A1(Baf A1) reversed the decrease of NOX4 under MOO treatment, indicating MOO decreased NOX by protease-mediated protein degradation (Figure 3H). In addition, as NOX4 is a key member of the NOX family, MOO-mediated NOX4 reduction attenuates the overall NOXs activity in a dose-dependent manner (Figure 3I). The above data demonstrate that NOX4 might be a potential target of MOO for ameliorating oxidative stress and myocardial ischemia.

### 2.4 MOO reduced oxidative stress injury and ferroptosis and promoted GPX4 mitochondrial translocation by inhibiting NOX4

**FIGURE 4:**
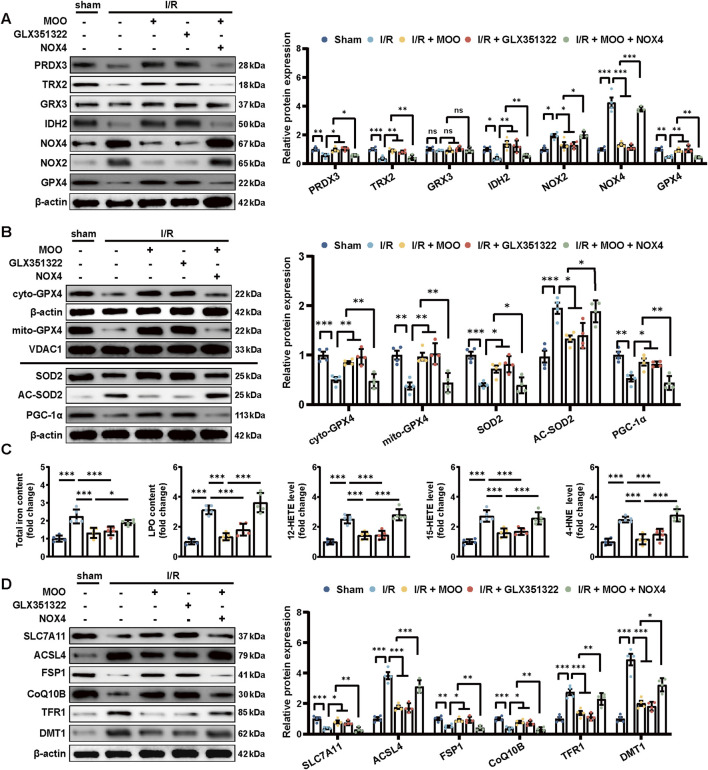
MOO pretreatment attenuated oxidative stress and inhibited ferroptosis by downregulating NOX4. **(A)** Western blot analysis of the levels of oxidative stress-related proteins in heart tissues. **(B)** Western blot analysis of the mitochondrial GPX4 (mito-GPX4) expression, cytoplasmic GPX4 (cyto-GPX4) expression, as well as cellular SOD2, acetylated SOD2 (AC-SOD2) and PGC-1α expression. **(C)** The contents of total iron and LPO and the levels of 12-HETE, 15-HETE, and 4-HNE in heart tissues. **(D)** Western blot analysis of SCL7A11, ACSL4, FSP1, CoQ10B, TFR1 and DMT1 protein expression in heart tissues. Data are expressed as the mean ± standard error (SE) and analysed by one-way ANOVA followed by Tukey’s multiple comparisons test. *P < 0.05. **P < 0.01. ***P < 0.001. ns, not significant. Four to six biological replicates were included in the experiment. (I/R, ischemia-reperfusion injury; LPO, lipid peroxidation; 12-HETE,12-hydroxyeicosatetraenoic acid; 15-HETE, 15-hydroxyeicosatetraenoic acid; 4-HNE, 4-Hydroxynonenal; ACL4, acyl-CoA synthetase long-chain family member 4; FSP1, ferroptosis suppressor protein 1; CoQ10B, protein CoQ10 homolog B; TFR1, transferrin receptor 1; DMT1, divalent metal transporter 1).

Given the inverse correlation between MOO treatment and NOX4 protein expression, we next sought to confirm whether MOO alleviated the antioxidative stress by targeting NOX4. We overexpressed NOX4 and applied NOX4-specific inhibitor GLX351322 (Supplementary Figure S2A). The results suggested that, compared with the sham group, the protein levels of PRDX3, TRX2, IDH2, and GPX4 protein were decreased, and the levels of NOX2 protein were increased in the I/R group, whereas these changes were significantly reversed with the addition of MOO or the NOX4-specific inhibitor GLX351322 (Figure 4A). In addition, the inhibitory effect of MOO on oxidative stress was significantly abolished by NOX4 overexpression, suggesting that MOO reduced myocardial oxidative stress by targeting NOX4 (Figure 4A).

In addition, we assessed whether MOO affected mitochondrial oxidative stress and improved mitochondrial function through NOX4. Given the prominent role of GPX4 in protecting cardiomyocyte mitochondria (Ahola and Langer, 2024), we examined the specific distribution of GPX4 in mitochondria. The results suggested that I/R injury triggered a significant reduction in GPX4 expression and its mitochondrial distribution; however, pretreatment with MOO or GLX351322 significantly improved GPX4 expression and promoted its translocation to the mitochondria (Figures 4A, B; Supplementary Figure S2B). These effects could be inhibited by NOX4 overexpression (Figures 4A, B; Supplementary Figure S2B). These data further demonstrate that MOO mediated the protective effects on mitochondrial oxidative stress by inhibiting NOX4 expression. To further evaluate whether MOO could improve mitochondrial function by targeting NOX4, the protein expression levels of SOD2, AC-SOD2, and PCG-1α were measured. NOX4-specific inhibitor GLX351322 and MOO showed similar effects in ameliorating SOD2 reduction, AC-SOD2 upregulation and PGC-1a suppression (Figure 4B). However, when NOX4 was overexpressed, the mitochondrial protection of MOO was abolished (Figure 4B). These data suggest that MOO reduced mitochondrial oxidative stress and improved mitochondrial function by targeting NOX4.

Considering the above results and the close association of GPX4 to ferroptosis (Tadokoro et al., 2020), we tested the potential effects of MOO on ferroptosis. I/R injury significantly exacerbated ferroptosis as indicated by elevated total iron and LPO content, along with increased levels of 12-HETE, 15-HETE, and 4-HNE adducts (Figure 4C). However, MOO or GLX351322 significantly ameliorated ferroptosis, but the transfection of NOX4 attenuated the benefits of MOO (Figure 4C). To further define the protective effect of MOO on ferroptosis, Western blotting was performed on ferroptosis-related signalling. I/R stress aggravated ferroptosis in myocardial tissue, as demonstrated by the elevated protein expression of Acyl-CoA synthetase long-chain family 4 (ACSL4), transferrin receptor 1 (TFR1), and divalent metal transporter 1 (DMT1), along with the decreased expression of solute carrier family 7 member 11 (SLC7A11), FSP1, and Coenzyme Q10B (CoQ10B). However, MOO or GLX351322 pretreatment attenuated this trend. As expected, the overexpression of NOX4 counteracted the ferroptosis-inhibitory effects of MOO (Figure 4D). Together, these findings suggest that MOO attenuates myocardial I/R injury potentially via the inhibition of mitochondrial oxidative stress and ferroptosis by targeting NOX4.

### 2.5 Protective effects of MOO against H/R-induced cardiomyocyte oxidative stress through NOX4 inhibition

**FIGURE 5:**
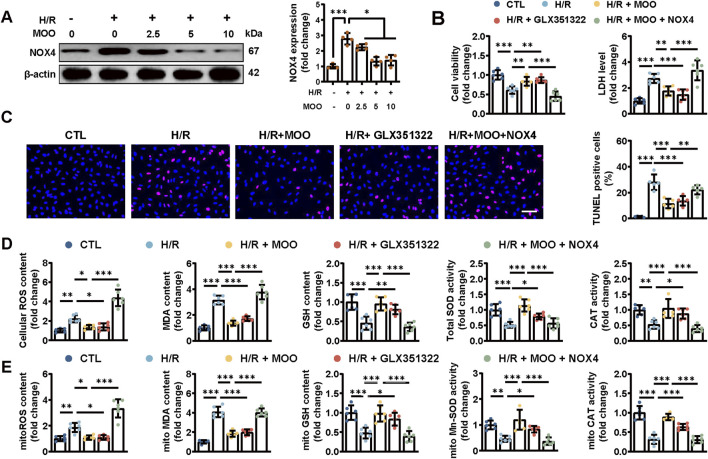
MOO pretreatment alleviated oxidative stress by NOX4 expression in cardiomyocytes after H/R injury. **(A)** NOX4 expression was detected by Western blot analysis with increased MOO dose under H/R injury. **(B)** Cell viability and LDH content in NMVCs. **(C)** Representative images and quantification of TUNEL staining. Scale bars = 40 μm. **(D)** Quantitative analysis of cellular oxidative stress markers. **(E)** Quantitative analysis of mitochondrial oxidative stress markers. Data are expressed as the mean ± standard error (SE) and analysed by one-way ANOVA followed by Tukey’s multiple comparisons test. *P < 0.05. **P < 0.01. ***P < 0.001. Four to six biological replicates were included in the experiment. (H/R, hypoxia-reoxygenation injury; MOO, *Morinda officinalis* oligosaccharides; NOX4, NADPH Oxidase 4; LDH, lactate dehydrogenase; NMVCs, newborn murine ventricular cardiomyocytes).

To gain a more detailed understanding of the antioxidant properties of MOO, we established an *in vitro* H/R model. MOO (2.5, 5 and 10 mg/mL) significantly enhanced cell viability, decreased LDH release and suppressed NOX4 protein expression under H/R condition (Figure 5A; Supplementary Figure S2C). Notably, the highest effects of MOO reached at a dose of 5 mg/mL, which was thereby selected for subsequent *in vitro* experiments. In addition, MOO also reduced the percentage of TUNEL-positive cells, supporting its pro-survival role in H/R-induced cardiomyocyte apoptosis (Figure 5C). These beneficial effects were also gained following GLX351322 pretreatment, whereas NOX4 overexpression counteracted the beneficial effects of MOO (Figures 5B, C; Supplementary Figure S2D). Furthermore, MOO or GLX351322 pretreatment alleviated cellular and mitochondrial oxidative stress, as shown by the decreased contents of cellular and mitochondrial ROS and MDA and the increased activities of total SOD, mitochondrial Mn-SOD, cellular and mitochondrial GSH and CAT (Figures 5D, E). However, overexpression of NOX4 abolished these MOO-offered protective effects (Figures 5D, E). Together, these data confirm that MOO attenuates cellular and mitochondrial oxidative stress in H/R-induced cardiomyocytes by inhibiting NOX4 expression.

### 2.6 Protective effects of MOO against H/R-induced cardiomyocyte ferroptosis through NOX4 inhibition

**FIGURE 6:**
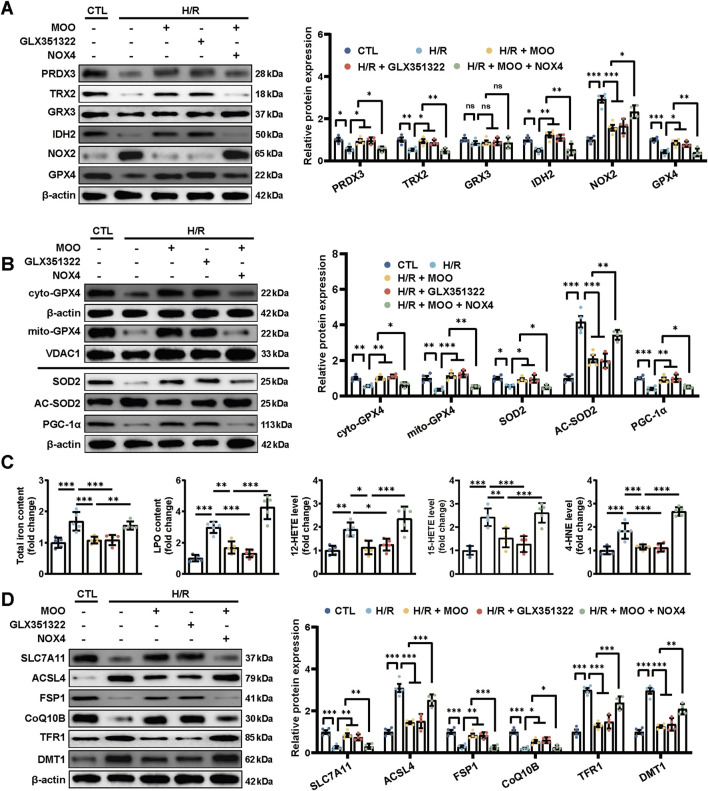
MOO pretreatment inhibited oxidative stress and ferroptosis by downregulating NOX4 in cardiomyocytes after H/R injury. **(A)** Western blot analysis of the levels of oxidative stress-related proteins in heart tissues. **(B)** Western blot analysis of the mitochondrial GPX4 (mito-GPX4) expression, cytoplasmic GPX4 (cyto-GPX4) expression, as well as cellular SOD2, acetylated SOD2 (AC-SOD2) and PGC-1α expression. **(C)** The contents of total iron and LPO and the levels of 12-HETE, 15-HETE, and 4-HNE in cardiomyocytes. **(D)** Western blot analysis of ferroptosis markers in cardiomyocytes. Data are expressed as the mean ± standard error (SE) and analysed by one-way ANOVA followed by Tukey’s multiple comparisons test. *P < 0.05. **P < 0.01. ***P < 0.001. ns, not significant. Four to six biological replicates were included in the experiment. (H/R, hypoxia-reoxygenation injury; LPO, lipid peroxidation; 12-HETE, 12-hydroxyeicosatetraenoic acid; 15-HETE, 15-hydroxyeicosatetraenoic acid; 4-HNE, 4-Hydroxynonenal).

To provide additional evidence that MOO affects oxidative stress levels by targeting NOX4 in cardiomyocyte, we overexpressed NOX4 and evaluated the expression levels of a range of oxidative stress-related proteins. The results showed that pretreatment with MOO or GLX351322 increased the expression levels of PRDX3, TRX2, IDH2 and GPX4 and decreased the expression levels of NOX2 in H/R-injured cardiomyocytes (Figure 6A). However, ADV-NOX4 transfection almost completely blocked these benefits of MOO treatment (Figure 6A). Moreover, MOO reduced mitochondrial oxidative stress and improved mitochondrial function by suppressing NOX4, as demonstrated by the increased mtDNA copy number, improved protein expression of SOD2, PGC-1α, and mitoGPX4, along with a decline in AC-SOD2 protein level, consistent with the *in vivo* results (Figure 6B; Supplementary Figures S2E, F). These data further support the beneficial effects of MOO on ameliorating oxidative stress and mitochondrial injury by suppressing NOX4.

Furthermore, pretreatment with MOO inhibited H/R-induced cardiomyocyte ferroptosis by suppressing NOX4 expression, as indicated by the decreases in the contents of total iron and LPO, as well as the levels of 12-HETE, 15-HETE, and 4-HNE (Figure 6C). Similarly, Western blotting confirmed the role of MOO in inhibiting ferroptosis through inhibition of NOX4 expression, as evidenced by increased protein levels of SLC7A11, FSP1, and CoQ10B and reduced levels of ACSL4, TFR1, and DMT1 (Figure 6D).

### 2.7 Mitochondrial GPX4 overexpression attenuated NOX4-induced cardiomyocyte injury under H/R condition

**FIGURE 7:**
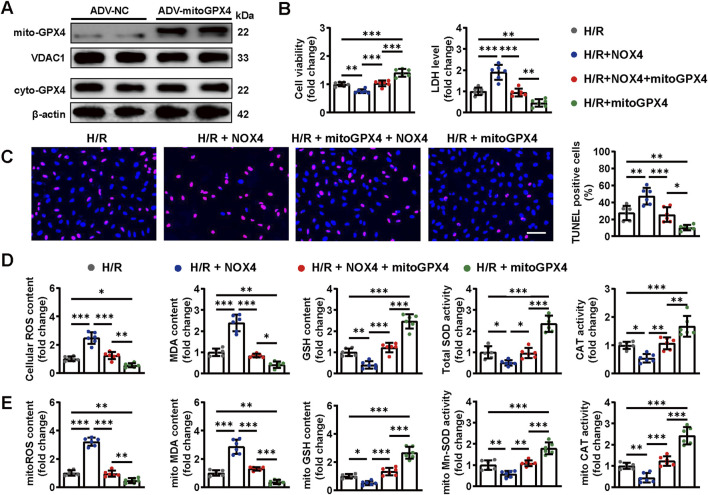
Mitochondrial GPX4 overexpression attenuated NOX4-induced cardiomyocyte injury under H/R condition. **(A)** mitoGPX4 transfection efficiencies were determined by Western blot analysis. **(B)** Cell viability and LDH content were determined in cardiomyocytes. **(C)** Representative images and quantitative analysis of TUNEL staining. Scale bars = 40 μm. **(D)** Quantitative analysis of cellular oxidative stress markers. **(E)** Quantitative analysis of mitochondrial oxidative stress markers. Data are expressed as the mean ± standard error (SE) and analysed by one-way ANOVA followed by Tukey’s multiple comparisons test. *P < 0.05. **P < 0.01. ***P < 0.001. Four to six biological replicates were included in the experiment. (H/R, hypoxia-reoxygenation injury; MOO, *Morinda officinalis* oligosaccharides; NOX4, NADPH Oxidase 4; LDH, lactate dehydrogenase).

We confirmed that MOO promoted mitochondrial translocation of GPX4 and improved oxidative stress by targeting NOX4 under H/R condition. Subsequently, an ADV-mediated mitochondrial-specific GPX4 (mitoGPX4) overexpression model was established to investigate whether the NOX4/mitoGPX4 pathway mediated the protective effects of MOO. The transfection efficiency of mitoGPX4 was assessed by Western blotting, confirming successful mitochondria-specific overexpression (Figure 7A). As shown above, the NOX4 overexpression significantly suppressed cell viability, elevated LDH levels, and increased the number of TUNEL-positive cells upon H/R injury, whereas these effects were evidently alleviated by mitoGPX4 overexpression (Figures 7B, C).

Furthermore, NOX4 overexpression aggravated cellular and mitochondrial oxidative stress under H/R condition, as indicated by increased contents of cellular and mitochondrial ROS and MDA, as well as reduced cellular and mitochondrial SOD, GSH and CAT. However, this oxidative stress caused by NOX4 overexpression were largely abolished by mitoGPX4 (Figures 7D, E). These findings confirm the crucial roles of the NOX4/mitoGPX4 pathway in cardiomyocytes oxidative stress under H/R condition.

### 2.8 Mitochondrial GPX4 overexpression attenuated NOX4-induced cardiomyocyte oxidative stress and ferroptosis under H/R condition

**FIGURE 8:**
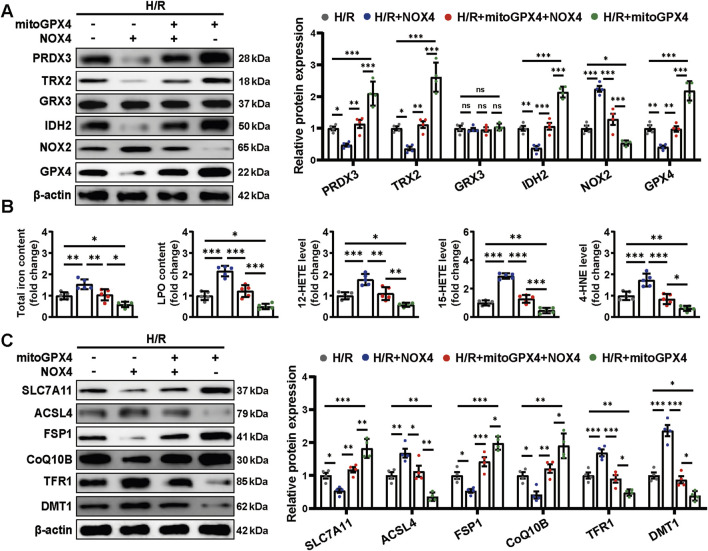
Mitochondrial GPX4 overexpression attenuated NOX4-induced cardiomyocyte oxidative stress and ferroptosis under H/R condition. **(A)** Western blot analysis of the levels of oxidative stress-related proteins in cardiomyocytes. **(B)** The content of total iron and LPO and the levels of 12-HETE, 15-HETE, and 4-HNE in cardiomyocytes. **(C)** Western blot analysis of ferroptosis markers in cardiomyocytes. Data are expressed as the mean ± standard error (SE) and analysed by one-way ANOVA followed by Tukey’s multiple comparisons test. *P < 0.05. **P < 0.01. ***P < 0.001. ns, not significant. Four to six biological replicates were included in the experiment. (H/R, hypoxia-reoxygenation injury; LPO, lipid peroxidation; 12-HETE, 12-hydroxyeicosatetraenoic acid; 15-HETE, 15-hydroxyeicosatetraenoic acid; 4-HNE, 4-Hydroxynonenal).

NOX4 overexpression further reduced the expression of antioxidative stress-related proteins upon H/R injury, the effects of which were largely reversed by mitoGPX4 overexpression (Figure 8A). Meanwhile, mitochondrial injuries were exacerbated accordingly by NOX4 overexpression, whereas these effects were counteracted by mitoGPX4 overexpression (Supplementary Figures S3A, B).

**FIGURE 9:**
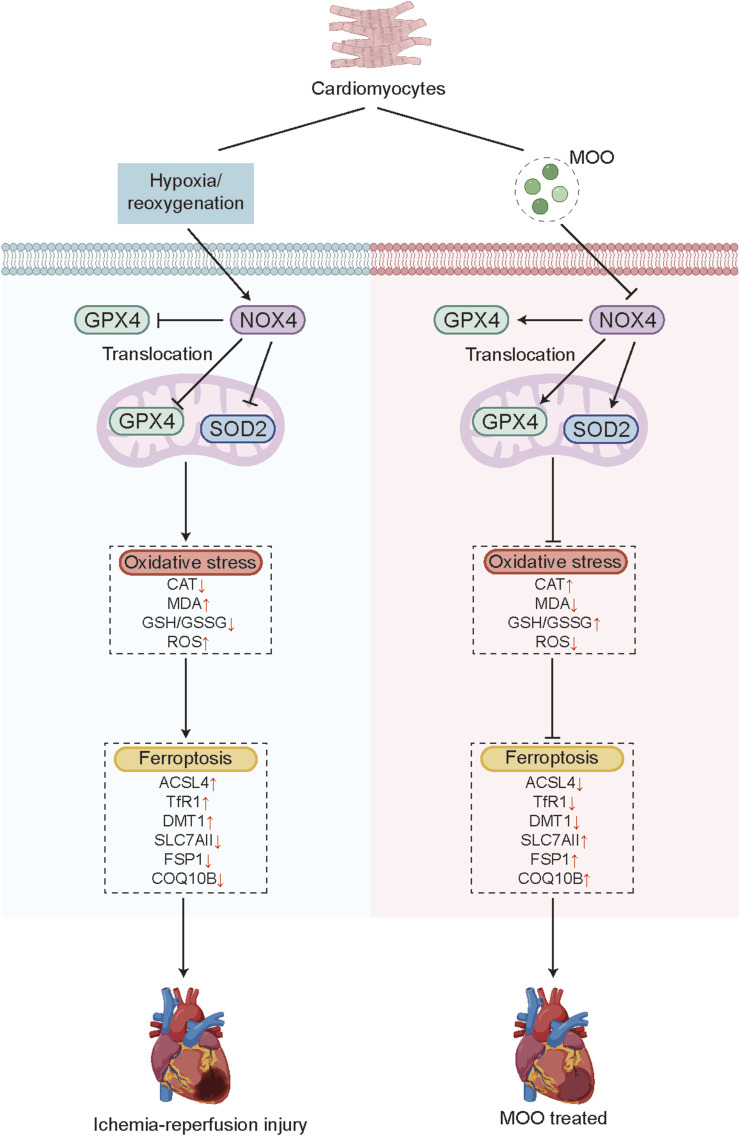
Model figure. MOO attenuates oxidative stress and ferroptosis in cardiomyocytes by NOX4/mitoGPX4 pathways under cardiac I/R injury.

In addition, we examined the NOX4/mitoGPX4 pathway on ferroptosis. NOX4 overexpression increased the total iron and LPO contents, as well as levels of 12-HETE, 15-HETE, and 4-HNE (Figure 8B). These alterations were reversed by mitoGPX4 overexpression (Figure 8B). Correspondingly, mitoGPX4 overexpression decreased the protein levels of ACSL4, TFR1, and DMT1 and increased the expression of SLC7A11, FSP1, and CoQ10B under NOX4 overexpression (Figure 8C). Together, these findings suggest a potential role of the NOX4/mitoGPX4 pathway in MOO-mediated cardioprotection against ferroptosis under H/R condition (Figure 9).

## 3 Discussion

In this study, we demonstrated that oxidative stress and ferroptosis occur in cardiac I/R injury and that MOO pretreatment confers resistance to oxidative stress and ferroptosis, thereby alleviating cardiac I/R injury. More importantly, NOX4 was identified as a downstream target of MOO. The beneficial effects of MOO on myocardial I/R injury were, at least in part, attributed to increased SOD2 activity and the promotion of GPX4 mitochondrial translocation by inhibiting NOX4 (summarized in Figure 9). These findings collectively suggest that suppressing ferroptosis and oxidative stress are important potential mechanisms for the cardioprotective benefits of MOO treatment on myocardial I/R injury.

ROS act as a double-edged sword; low levels of ROS are beneficial, but overaccumulation leads to DNA damage, lipid peroxidation, and protein oxidation. Mitochondria serve as a major source of ROS production during myocardial I/R injury. Mitochondrial ROS at physiological level activates cell survival programs and enhances tolerance to ischemia; conversely, the overproduction of mitochondrial ROS results in ROS-induced ROS release, exacerbating oxidative stress and promoting mitochondrial permeability transition (Zorov et al., 2014). This in turn triggers mitochondrial swelling and mPTP opening, which accentuate cell death during myocardial I/R injury (Mendoza et al., 2024). A growing body of evidence supports the beneficial role of mitochondria-directed antioxidants in reducing myocardial I/R injury (Walters et al., 2012). In addition, emerging evidence demonstrates the indispensable role of mitochondria in regulating the initiation and execution of ferroptosis, defined as mitochondria-dependent ferroptosis, primarily due to excessive ROS production (Chen et al., 2023). In the current study, we confirmed mitoROS overproduction and LPO accumulation after myocardial I/R injury, accompanied by reduced GSH content and GPX4 expression, which support the significant role of mitochondrial oxidative stress and ferroptosis in the development of myocardial I/R injury. In addition, our data further support the inhibitory effect of MOO on oxidative stress and ferroptosis after myocardial I/R injury, at least partly attributed to the improvement of mitochondrial function.

NOX4, a member of the NADPH oxidase family, is widely expressed in the cardiomyocyte (Lassègue et al., 2012). Unlike the other NOX isoforms, NOX4 is s constitutively activated at moderate level (Leto et al., 2009). The fundamentally elevation of NOX4 induces minimal level of ROS which subsequently active HIF-α and contributes to myocardial adaptation during I/R injury and pressure-overload (Matsushima et al., 2013). However, the beneficial effects were carried out only by minimal level of ROS derived from NOX4, and might be exerted in a cell and organelle-specific manner. Previous studies revealed that during I/R injury, NOX4 in the endoplasmic reticulum (ER) is potentially protective through maintaining autophagy and ER-mitochondria contact sites function, whereas mitochondrial NOX4 causes ROS-induced mitochondrial dysfunction (Matsushima and Sadoshima, 2022). Therefore, with the poor therapeutical outcome yielded from ROS manipulation during I/R, selective inhibition of NOX4 in mitochondria was raised as the promising strategy. The present studies confirmed the possible detrimental effects of total NOX4 upregulation on cardiac I/R injury and suggests that MOO treatment may ameliorate myocardial I/R injury largely by inhibiting NOX4.

GPX4, a unique antioxidant enzyme, converts lipid hydroperoxides to nontoxic lipid alcohols, effectively preventing ferroptosis. It exists in three specific subcellular locations: the cytosol, mitochondria, and nucleus. In the present study, we found that GPX4 levels in either the cytosol or mitochondria were reduced in both I/R-injured myocardium and H/R-injured cardiomyocytes. Recent studies suggested that mitoGPX4 fulfilled important roles in ferroptosis resistance of cardiomyocytes. They found that ferroptosis induced by doxorubicin and trastuzumab was triggered by mitochondria, whereas mitoGPX4 effectively suppresses lipid peroxides and ferroptosis (Ye et al., 2023; Tadokoro et al., 2020). In addition, miotGPX4 overexpression has been reported to improve cardiac contractile function following mice myocardial I/R injury (Dabkowski et al., 2008). Moreover, in cultured cardiomyocytes under H/R condition, overexpression of mitochondrial GPX4 protects cells from oxidative stress, decreases the release of CK and LDH, preserves electron transport chain complex function, and inhibits apoptosis (Hollander et al., 2003). Our results were consistent with these studies. With the mitoGPX4 overexpression, cellular and mitochondrial oxidative stress induced by H/R were alleviated, together with mitigated ferroptosis. Hence, the current data confirm that the suppression of mitoGPX4 level is critical for I/R-induced ferroptosis and suggest that the protective role of MOO on myocardial I/R injury is partly due to the upregulation of mitochondrial GPX4.

In addition to recently emerging evidence of the benefits of MOO in protecting endothelial cells from H/R-induced injury, our work provides further evidence of the benefits of MOO on myocardial I/R injury by relieving oxidative stress and ferroptosis. However, some limitations in our study should be considered. First, although we have demonstrated the downregulation of NOX4 expression promoted the mitochondrial translocation of GPX4, the detailed mechanism requires further exploration. Also, it remains to be established whether MOO treatment confers similar cardioprotective effects in other cardiovascular condition, such as diabetic cardiomyopathy and heart failure.

In conclusion, we demonstrated the cardioprotective effects of MOO on myocardial I/R injury. Mechanistic studies showed that MOO effectively suppressed NOX4, enhanced SOD2 activity, and promoted GPX4 translocation to mitochondria, therefore, attenuating oxidative stress and ferroptosis in myocardial I/R injury. These data suggest that the NOX4/SOD2/mitoGPX4 pathway may be a promising therapeutic target for I/R-induced myocardial injury. Inhibiting NOX4 levels and enhancing mitochondrial GPX4 levels may be beneficial for the minimization of myocardial I/R injury.

## 4 Materials and methods

### 4.1 Animals

All animal experiments were approved by the Research Ethics Committee of Nanjing Medical University (Jiangsu, China) and strictly adhered to the National Institutes of Health Guide for the Care and Use of Laboratory Animals. Six-week-old male C57BL/6 mice purchased from M.Q. MICROBE Co., Ltd. (Suzhou, China) were used in this study. Mice were housed in laboratory animal facilities with a 12 h light/dark cycle and were provided food and water *ad libitum*.

The surgical procedures for cardiac I/R injury were performed as previously described (Li S. et al., 2021). The left anterior descending (LAD) artery was ligated using a 6-0 silk suture with a slipknot for 45 min followed by a 24 h reperfusion period. Sham-operated mice underwent the same surgical procedures except that the suture was not tied.

To examine the functional role of MOO, mice were orally administered MOO (Peking Tong Ren Tang Pharmaceutical Ltd. Co., China) at a dosage of 100 mg/kg/day for 2 weeks before I/R injury, and the last dosage was given 6 h before I/R surgical procedures (Zhang et al., 2022; Li Z. et al., 2021). To inhibit NADPH Oxidase 4 (NOX4) *in vivo*, 3.8 mg/kg GLX351322 (MCE, United States) was dissolved in 0.5% dimethyl sulfoxide (DMSO) and administered via intraperitoneal injection 1 h before I/R injury. Intramyocardial injection was performed 3 days before cardiac I/R injury for NOX4 overexpression. After anesthesia, the needle was inserted into the anterior wall of the left ventricle for the injection of adenoviral containing NOX4 gene (ADV-NOX4) at a dose of 1.0 × 10^9^ vector genomes/heart (Wu R. et al., 2024). The transfection efficiency was determined using Western blotting 72 h after transfection.

### 4.2 Echocardiography analyses of cardiac function

Transthoracic echocardiography was performed with a Vevo 2100 Ultrasound System (VisualSonics, Toronto, Ontario, Canada) following I/R injury and corresponding control surgical procedures. Mice were anaesthetised with 2% isoflurane, and heart rates were maintained at 400 beats/min. Echocardiography was applied to directly evaluate the left ventricular ejection fraction (LVEF) and left ventricular fractional shortening (LVFS). After echocardiography, the mice were immediately euthanized to collect serum and heart tissue samples.

### 4.3 Histopathological analysis

Heart tissue samples were fixed in 4% paraformaldehyde, gradually dehydrated, embedded in paraffin, and cut into 5-micron-thick sections. After staining with haematoxylin and eosin (HE), the cardiac sections were examined via a light microscopy.

### 4.4 Measurement of myocardial injury markers

Blood samples, collected by orbital bleeding into heparinized tubes, were allowed to clot for 30 min and then centrifuged at 3,000 rpm for 10 min at 4°C to obtain serum samples. Myocardial injury markers, including lactate dehydrogenase (LDH), creatine kinase MB (CK-MB), cardiac troponin (cTnI), and myoglobin (Myo), as well as creatinine (Cr), were measured using serological index kits or mouse-specific ELISA kits (Nanjing Jiancheng Bio, China) based on the manufacturer’s instructions. The catalogue number of assay kits used in this study was provided in Supplementary Table S1.

### 4.5 Cell culture and treatment

Newborn murine ventricular cardiomyocytes (NMVCs) were isolated from neonatal 1-day-old mouse myocardial tissue as previously described and were cultured in DMEM (Invitrogen, United States) supplemented with 10% foetal calf serum in an incubator at 37°C with 5% CO_2_ (Shi et al., 2021). NMVCs were washed twice with PBS to remove culture medium and incubated with fresh ischemia Esumi buffer. Cells were then transferred to a hypoxia chamber (Billups-Rothenberg, San Diego, United States) under 1% O_2_ (94% N_2_, 5% CO_2_) for 12 h. Reoxygenation was performed by replacing the ischemic buffer with fresh medium and returning cells to normoxic condition for 6 h to simulate cardiomyocytes H/R injury *in vitro* (Shi et al., 2021).

For MOO pretreatment, NMVCs were treated with MOO at a concentration of 5–10 mg/mL for 12 h before hypoxia-reoxygenation (H/R) injury (Zhu et al., 2022). To overexpress NOX4, NMVCs were treated with ADV-NOX4 for 24 h and the transfection efficiency was determined using Western blotting 48 h after transfection (Fan et al., 2020). Mitochondrial specific overexpression of GPX4 was induced in the same way, except a mitochondrial transit peptide was integrated into the N-terminus of GPX4, denoted as mitoGPX4 (Tadokoro et al., 2020; Mao et al., 2021).

### 4.6 Drug affinity responsive target stabilization assay (DARTS)

A DARTS assay was conducted as previously described (Liu et al., 2024). NMVCs were lysed on ice with M-PER™ (Thermo Scientifc) for 30 min, after which the cell lysate was quantified by Bradford protein assay kit (Beyotime, China) and diluted to 5 mg/mL. DMSO control or 10 μM MOO diluted in TNC buffer (50 mmol/L Tris, 50 mmol/L NaCl, and 10 mmol/L CaCl2, pH 7.4) was added to the cell lysate. The samples were mixed gently and incubated overnight at 4°C for sufficient ligand–protein binding. Subsequently, half of the lysates were digested with pronase for 30 min were, and the other half served as loading control. Finally, the above samples were boiled for 10 min with loading buffer, separated by SDS-PAGE and stained with Coomassie or quantified by Western blotting.

### 4.7 Measurement of oxidative damage biomarkers

Protein samples were extracted from board zone of cardiac tissues, cardiomyocytes, or isolated mitochondria via lysis and centrifugation. A BCA Protein Assay Kit (Beyotime, China) was used to quantify the protein levels. The malondialdehyde (MDA) content was measured by a malondialdehyde assay kit (Nanjing Jiancheng Bio, China). The glutathione (GSH) and oxidized glutathione (GSSG) concentrations were determined using GSH and GSSG Assay Kits (Beyotime, China). The enzyme activities of catalase (CAT) and thioredoxin reductase (TRXR) were quantified using CAT and TRXR test kits (Nanjing Jiancheng Bio, China). The content of sulfhydryl (SH) was quantified using an SH content detection kit, and the nonprotein SH (NPSH) concentration was determined following the Sedlak and Lindsay (1968) method (Sanchez-Moya et al., 2022). Superoxide dismutase (SOD) and Manganese-SOD (Mn-SOD) activities were measured by Cu/Zn-SOD and Mn-SOD assay kits with WST-8 (Beyotime, China). Nicotinamide adenine dinucleotide phosphate (NADPH) oxidases (NOXs) activity was measured by a CheKine™ Micro NADH Oxidase (NOX) Assay Kit (Abbkine, China). The abovementioned assays were performed according to the instructions and standardized to protein concentrations. The catalogue number of assay kits used in this study was provided in Supplementary Table S1.

### 4.8 Measurement of 12-HETE, 15-HETE, and 4-HNE levels

To determine 12-hydroxyeicosatetraenoic acid (12-HETE) and 15-HETE levels, 12-HETE and 15-HETE ELISA kits (Abcam, United States) were used according to the product instructions. To detect 4-Hydroxynonenal (4-HNE), the OxiSelectTM 4-HNE Assay Kit (Cell Biolabs Inc., United States) was utilized based on the kit instruction manual. The catalogue number of assay kits used in this study was provided in Supplementary Table S1.

### 4.9 TUNEL staining

TUNEL staining was performed to determine apoptosis in the board zone and cultured cardiomyocytes using an *in situ* TUNEL Detection Kit (Roche, Switzerland) in accordance with the manufacturer’s protocol. DAPI (Beyotime, China) was used to stain the nuclei, and the samples were observed under a confocal microscope (Olympus FV3000, Japan).

### 4.10 Cell viability assay

Cell viability was measured using a CCK-8 assay (Epizyme, Cambridge, MA) according to the manufacturer’s instructions. Cardiomyocytes were cultured in 96-well plates with 100 μL of CCK-8 working solution per well and incubated at 37°C for 2 h. A microplate reader (Molecular Devices, PA) was used to read the absorbance at 450 nm.

### 4.11 ROS detection

Cellular ROS and mitochondrial ROS (mitoROS) were measured with an ROS staining kit (Beyotime, China) and a MitoSOX indicator (Invitrogen, CA). Cardiomyocytes were incubated with 5 μmol/L 2′-7′dichlorofluorescin diacetate or 5 μmol/L MitoSOX working solution for 20 min at 37°C under dark condition. The samples were observed under a confocal microscope (Olympus FV3000, Japan). ImageJ (version 1.53c; National Institutes of Health, Bethesda, MD) was used to quantify the mean fluorescence intensities of cellular ROS and mitoROS.

### 4.12 Iron content detection

Total iron content was measured by an iron assay kit (Abcam) according to the product instructions. Briefly, mouse myocardial tissue or cardiomyocytes in iron assay lysis buffers were rapidly homogenized and centrifuged at 16,000 g at 4°C for 10 min. The supernatant was then incubated with an iron reducer at 25°C for 30 min followed by incubation with iron probe buffer for 60 min at 25°C in the dark. A microplate reader (Molecular Devices) was used to read the absorbance at 593 nm.

### 4.13 Lipid peroxidation analysis

The lipid peroxidation (LPO) content was measured via a lipid peroxidation assay kit based on the manufacturer’s instructions (Nanjing Jiancheng Bio, China). In short, the chromogen reagent was combined with lipid peroxide and incubated at 45°C for 60 min to stabilize the chromophore. Readings were subsequently obtained using a microplate reader at 586 nm. The lactate dehydrogenase (LDH) release assay was conducted using a LDH Cytotoxicity Assay Kit (MedChemExpress, Monmouth Junction, China). All the above experiments were performed according to the manufacturer’s instructions.

### 4.14 Western blotting

Mitochondria and cytoplasm were isolated by a cell mitochondria isolation kit (Beyotime Biotechnology) according to the manufacturer’s instructions. Protein samples were extracted from tissues, cells, cytoplasm, and mitochondria using RIPA lysis buffer supplemented with phenylmethanesulfonyl fluoride and phosphatase inhibitors and then centrifuged at 12,000 rpm for 20 min at 4°C. After quantification with BCA protein assay kit (Beyotime), protein samples were separated in SDS-polyacrylamide electrophoresis gels and transferred to polyvinylidene difluoride membranes. After blocking, the membranes were incubated with the indicated primary antibodies overnight at 4°C, followed by incubation with horseradish peroxidase-conjugated secondary antibodies for 1 h at room temperature. Afterwards, protein bands were detected by electrochemiluminescence Western blotting substrate (Thermo Fisher Scientific), and the obtained images were analysed using ImageJ software (version 1.53c; National Institutes of Health). VDAC1 was used as loading control for mitochondrial fragment. β-actin was used as loading control for cytosolic fragment and total cell lysates. Detailed information on the primary antibodies used for Western blotting is shown in Supplementary Table S2.

### 4.15 Molecular docking

To explore the potential configurational interaction between MOO and NOX4, we obtained the chemical structure of MOO from PubChem (https://pubchem.ncbi.nlm.nih.gov/) and predicted protein structures of PRDX3, TRX2, IDH2, NOX2,NOX4 and GPX4 by AlphaFold3 software (https://alphafold.ebi.ac.uk/) (Jumper et al., 2021; Wang et al., 2012), respectively. Then a mock docking was performed using Schrödinger-Maestro (Version win2022-1). Binding score indicating the affinity for proteins and MOO was calculated, and the results was analysed by PLIP (https://plip-tool.biotec.tu-dresden.de/plip-web/plip/index) and visualized using pymol (Version 2.5).

### 4.16 Statistical analysis

Statistical analyses were performed using GraphPad Prism 9.0 (San Diego, CA). All data are presented as the mean ± standard deviation (SD). Student’s t-test and one-way ANOVA followed by Tukey’s test were performed for comparisons. P values <0.05 were considered significantly different.

## Data Availability

The datasets presented in this study can be found in online repositories. The names of the repository/repositories and accession number(s) can be found in the article/Supplementary Material.

## Supplementary material

SUPPLEMENTARY FIGURE S1
(A) Statistical analysis of left ventricular fractional shortening (LVFS) in indicated groups (n = 12). **(B)** The enzyme activities of CAT in mitochondria isolated from heart tissues. **(C, D)** Western blot analysis of SOD2, Ac-SOD2, and PGC-1α protein expression in heart tissues. Data are expressed as the mean ± standard error (SE) and analysed by one-way ANOVA followed by Tukey’s multiple comparisons test. **P* < 0.05, ***P* < 0.01, ****P* < 0.001 between sham and I/R groups. ^#^P < 0.05, ^##^P < 0.01, ^###^P < 0.001 between I/R and I/R + MOO groups. Four to twelve biological replicates were included in the experiment.

SUPPLEMENTARY FIGURE S2
(A) The overexpression efficiency of ADV-NOX4 was detected by Western blot analysis. **(B)** Statistical analysis of mitochondrial GPX4 (mito-GPX4) to cytoplasmic GPX4 (cyto-GPX4) ratio in indicated groups. **(C)** Cell viability and LDH content were detected in NMVCs with increased MOO doses under H/R injury. **(D)** Western blot analysis of NOX4 expression after GLX351322 treatment and ADV-NOX4 transfection. **(E)** Statistical analysis of mtDNA copy number in indicated groups. **(F)** Statistical analysis of mitochondrial GPX4 (mito-GPX4) to cytoplasmic GPX4 (cyto-GPX4) ratio in indicated groups. Data are expressed as the mean ± standard error (SE) and analysed by one-way ANOVA followed by Tukey’s multiple comparisons test. **P* < 0.05. ***P* < 0.01. ****P* < 0.001. Four to six biological replicates were included in the experiment.

SUPPLEMENTARY FIGURE S3
(A) Western blot analysis of cellular SOD2, acetylated SOD2 (AC-SOD2) and PGC-1α protein expression in indicated groups. **(B)** Statistical analysis of mtDNA copy number in indicated groups. Data are expressed as the mean ± standard error (SE) and analysed by one-way ANOVA followed by Tukey’s multiple comparisons test. **P* < 0.05. ***P* < 0.01. ****P* < 0.001. Four to six biological replicates were included in the experiment.
