# Supplementary material for: Morinda officinalis oligosaccharides attenuate mitochondria-associated ferroptosis via the NOX4/mitoGPX4 pathway in myocardial ischemia‒reperfusion injury
Source: Front Cell Dev Biol. 2025 May 26;13:1605513. doi: 10.3389/fcell.2025.1605513 (PMC12146387; doi:10.3389/fcell.2025.1605513)
Supplement: Supplementary file 1 [file Table1.docx]

Table S2 Primary antibodies used in western blots.

| Name | Manufacturer | Cat No. |
| --- | --- | --- |
| lactate dehydrogenase (LDH) | Nanjing Jiancheng Bio | A020-2-2 |
| creatine kinase MB (CK-MB) | Nanjing Jiancheng Bio | H197-1-1 |
| cardiac troponin (cTnI) | Nanjing Jiancheng Bio | H149-2-2 |
| myoglobin (Myo) | Nanjing Jiancheng Bio | H150-1-1 |
| creatinine (Cr) | Nanjing Jiancheng Bio | C011-2-1 |
| GLX351322 | MCE | HY-100111 |
| DMEM | Invitrogen | 11965092 |
| M-PER™ | Thermo Scientifc | 78505 |
| Bradford protein assay kit | Beyotime | P0006 |
| BCA Protein Assay Kit | Beyotime | P0012 |
| malondialdehyde assay kit | Nanjing Jiancheng Bio | A003-4-1 |
| GSH and GSSG Assay Kits | Beyotime | S0053 |
| Oxidized thioredoxin reductase (TrxR) test kit | Nanjing Jiancheng Bio | A119-1-1 |
| Catalase (CAT) assay kit | Nanjing Jiancheng Bio | A007-1-1 |
| SH content detection kit | Nanjing Jiancheng Bio | A063-2-1 |
| Cu/Zn-SOD and Mn-SOD assay kits | Beyotime | S0103 |
| CheKine™ Micro NADH Oxidase (NOX) Assay Kit | Abbkine | KTB1021 |
| 12(S)-HETE ELISA | Abcam | ab133034 |
| 15(S)-HETE ELISA | Abcam | ab133035 |
| OxiSelectTM 4-HNE Assay Kit | Cell Biolabs Inc. | STA-838 |
| TUNEL Detection Kit | Roche | 12156792910 |
| DAPI | Beyotime | P0131 |
| CCK-8 assay | Epizyme | CX001S |
| ROS staining kit | Beyotime | S0033S |
| MitoSOX indicator | Invitrogen | M36009 |
| iron assay kit | Abcam | ab83366 |
| lipid peroxidation assay kit | Nanjing Jiancheng Bio | A106-1-2 |
| LDH Cytotoxicity Assay Kit | MedChemExpress | HY-K1090 |
| electrochemiluminescence Western blotting substrate | Thermo Fisher Scientific | 34578 |
|  |  |  |
|  |  |  |
|  |  |  |
|  |  |  |
|  |  |  |
