# Supplementary figures and images for: Morinda officinalis oligosaccharides attenuate mitochondria-associated ferroptosis via the NOX4/mitoGPX4 pathway in myocardial ischemia‒reperfusion injury
Source: Front Cell Dev Biol. 2025 May 26;13:1605513. doi: 10.3389/fcell.2025.1605513 (PMC12146387; doi:10.3389/fcell.2025.1605513)

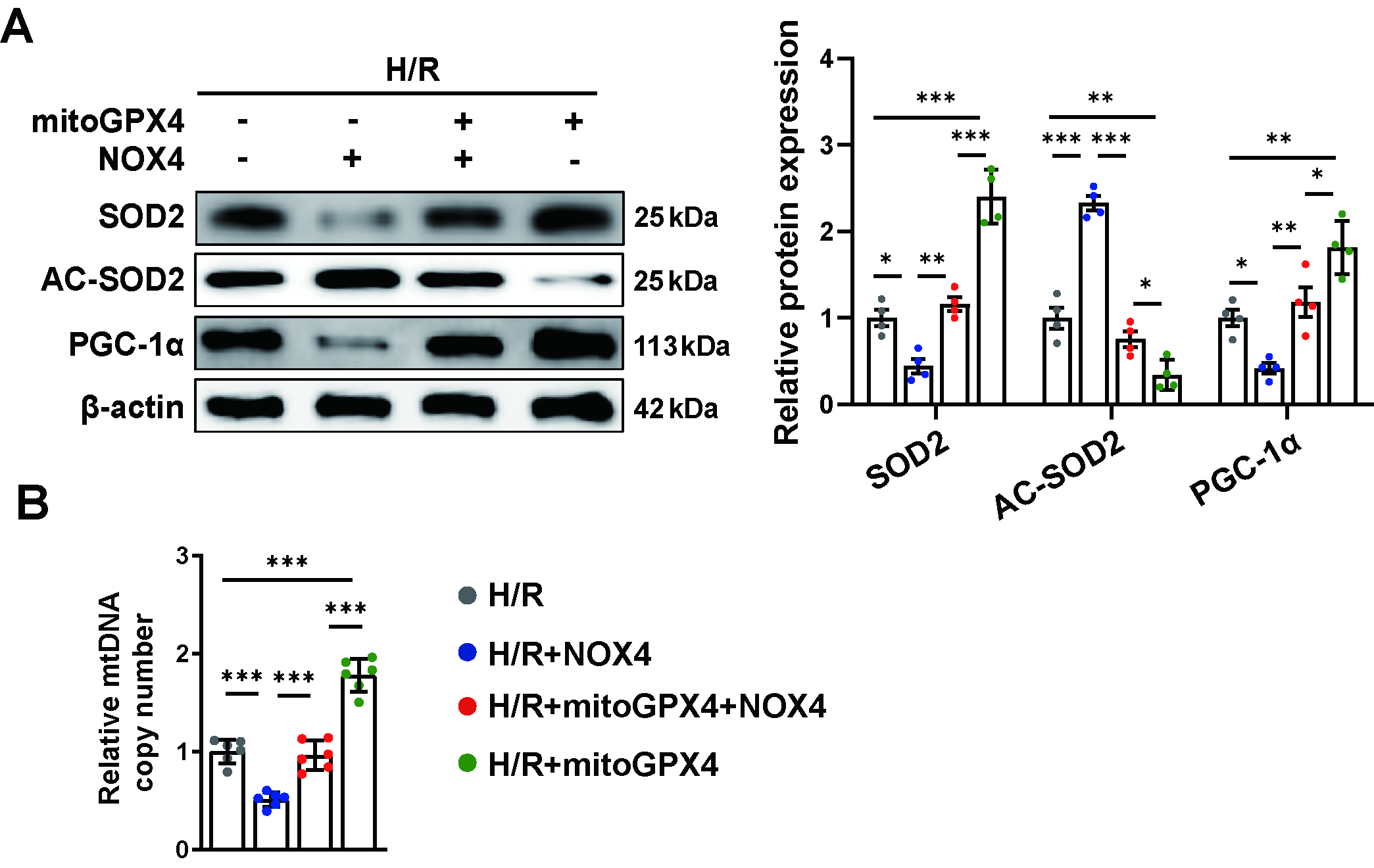

Supplement: Supplementary file 2 [file Image3.tif]

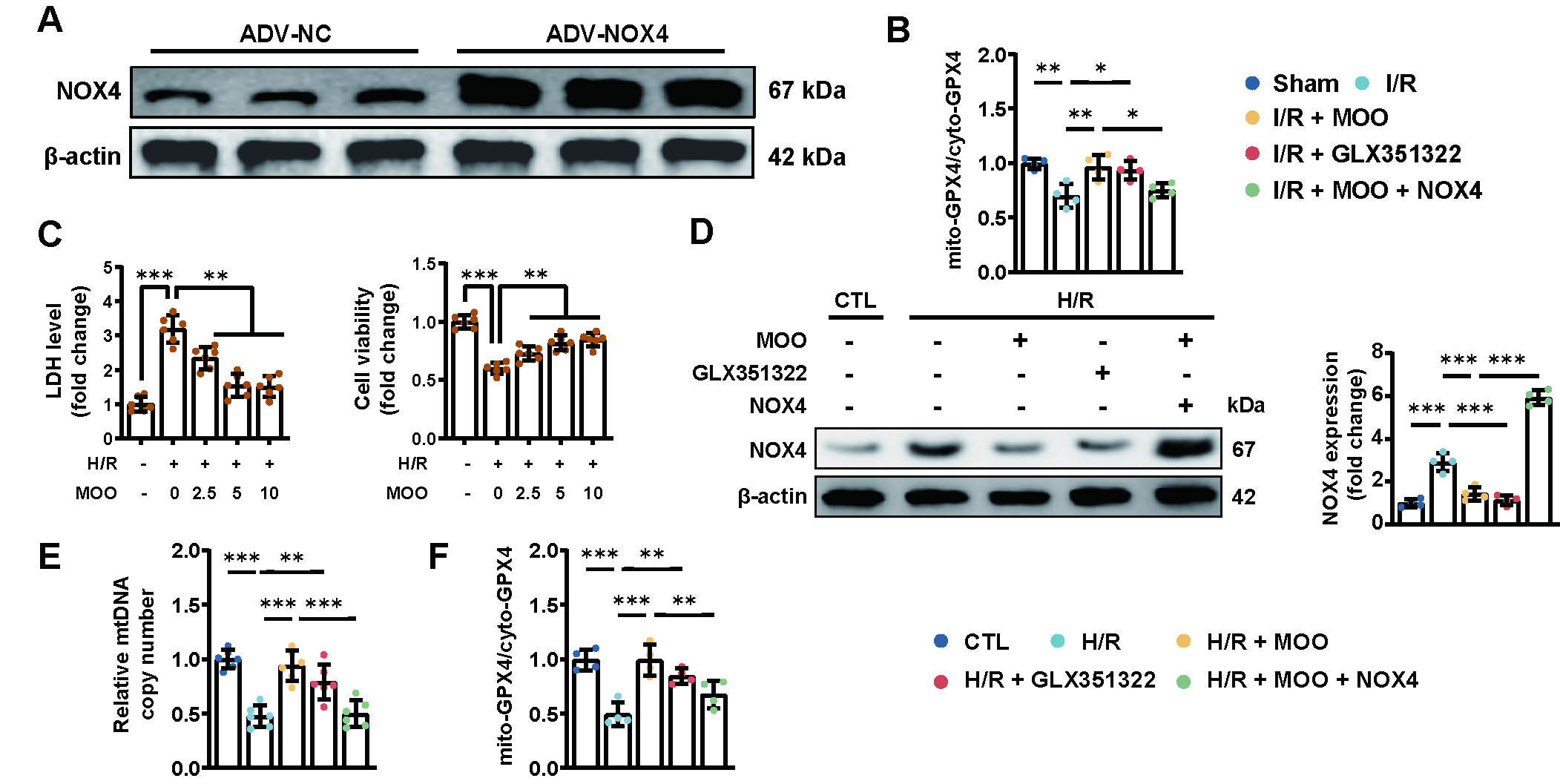

Supplement: Supplementary file 3 [file Image2.tif]

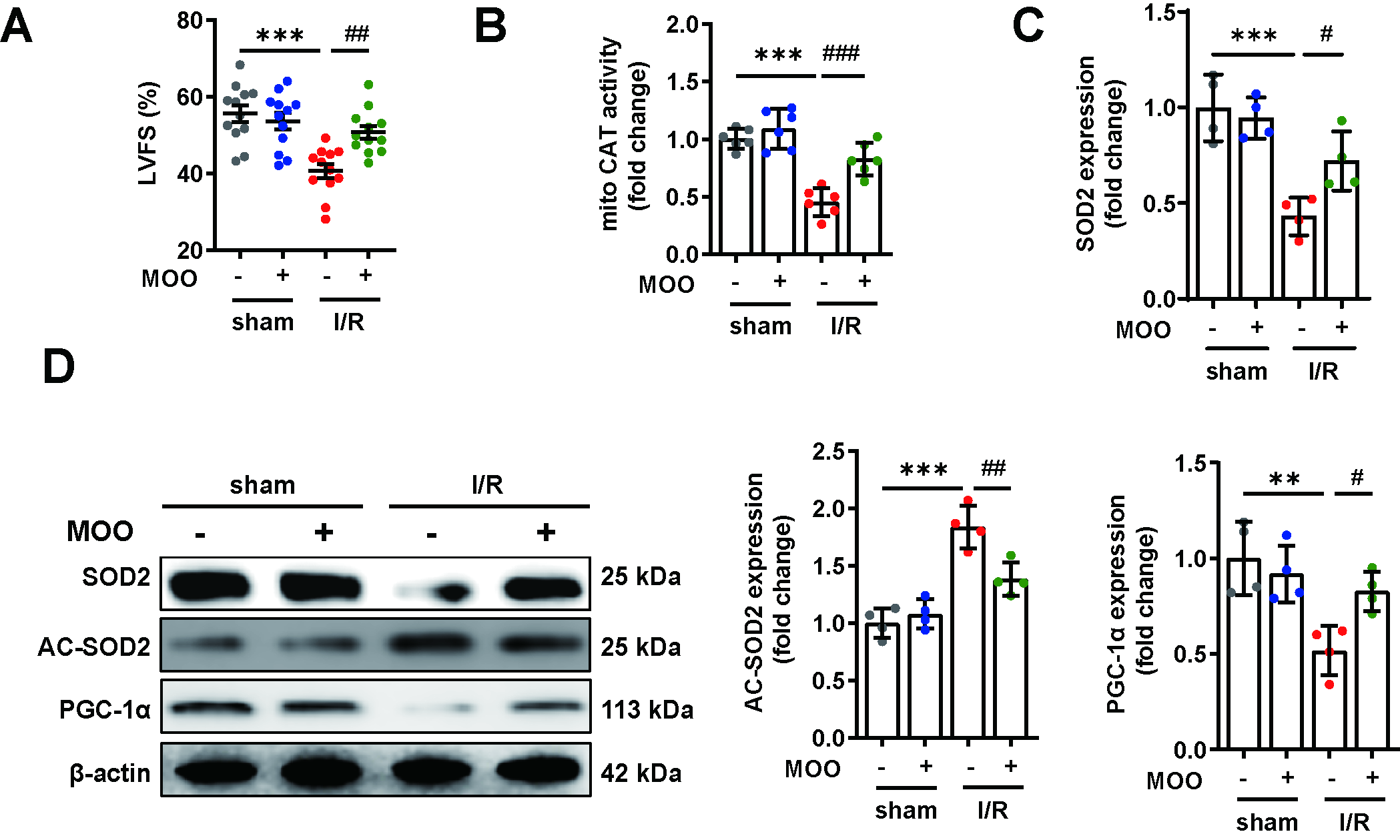

Supplement: Supplementary file 4 [file Image1.tif]
