# Supplementary material for: Morinda officinalis oligosaccharides attenuate mitochondria-associated ferroptosis via the NOX4/mitoGPX4 pathway in myocardial ischemia‒reperfusion injury
Source: Front Cell Dev Biol. 2025 May 26;13:1605513. doi: 10.3389/fcell.2025.1605513 (PMC12146387; doi:10.3389/fcell.2025.1605513)
Supplement: Supplementary file 5 [file Table2.docx]

Table S2 Primary antibodies used in western blots.

| Name | Manufacturer | Cat No. | Dilution | Reacts with: |
| --- | --- | --- | --- | --- |
| β-actin | Abcam | Ab8226 | 1:1000 | Mouse, Rat, Human |
| SOD2 | Abcam | Ab68155 | 1:1000 | Mouse, Rat, Human |
| AC-SOD2 | Abcam | Ab218529 | 1:1000 | Mouse, Rat, Human |
| GPX4 | Abcam | Ab125066 | 1:3000 | Mouse, Rat, Human |
| PGC-1α | Abcam | Ab191838 | 1:1000 | Mouse, Rat, Human |
| PRDX3 | Abcam | Ab73349 | 1:1000 | Mouse, Rat, Human |
| TRX2 | Abcam | Ab185544 | 1:10000 | Mouse, Human |
| GRX3 | Abcam | Ab226396 | 1:1000 | Mouse, Human |
| IDH2 | Abcam | Ab131263 | 1:1000 | Mouse, Rat, Human |
| NOX2 | Abcam | Ab310337 | 1:1000 | Mouse, Rat, Human |
| NOX4 | Abcam | Ab154244 | 1:1000 | Mouse, Rat, Human |
| VDAC1 | Abcam | Ab306581 | 1:1000 | Mouse, Rat, Human |
| SLC7A11 | Abcam | Ab175186 | 1:1000 | Mouse, Rat, Human |
| ACSL4 | Abcam | Ab155282 | 1:10000 | Mouse, Rat, Human |
| FSP1 | Abcam | Ab302673 | 1:1000 | Mouse, Rat, Human |
| CoQ10B | Affinity | DF13998 | 1:1000 | Mouse, Rat, Human |
| TFR1 | Abcam | Ab269513 | 1:5000 | Mouse, Rat, Human |
| DMT1 | Abcam | Ab55735 | 1:1000 | Mouse, Human |
|  |  |  |  |  |
|  |  |  |  |  |
|  |  |  |  |  |
|  |  |  |  |  |
|  |  |  |  |  |
|  |  |  |  |  |
|  |  |  |  |  |
|  |  |  |  |  |
|  |  |  |  |  |
|  |  |  |  |  |
